# Metallothionein expression in feline injection site fibrosarcomas

**DOI:** 10.1186/s12917-023-03604-5

**Published:** 2023-02-10

**Authors:** Mateusz Mikiewicz, Katarzyna Paździor-Czapula, Joanna Fiedorowicz, Michał Gesek, Iwona Otrocka-Domagała

**Affiliations:** Department of Pathological Anatomy, Faculty of Veterinary Medicine, University of Warmia and Mazury in Olsztyn, Oczapowskiego 13, 10-719, Olsztyn, Poland

**Keywords:** Feline injection site fibrosarcoma, Proliferation index, Ki67, Metallothionein, Immunohistochemistry

## Abstract

**Background:**

Feline injection site fibrosarcoma is an aggressive and infiltrative tumour arising in the background of chronic inflammation. The aim of this study was to evaluate the expression of metallothionein (I-II) in feline injection site fibrosarcomas and to assess its possible relationships with Ki67 index, inflammation score and tumour grade. The study included 40 feline fibrosarcomas, located in the common injection sites (i.e., interscapular area, thigh, flank), constituting archival diagnostic specimens collected between 2019–2020. Tumours were graded histologically according to the newly proposed soft-tissue sarcoma grading system in cats. Immunohistochemistry was performed to evaluate the expression of Ki67 and metallothionein in tumour cells.

**Results:**

The cytoplasmic and sometimes nuclear expression of metallothionein was observed in all tumours grade I, 66.67% of tumours grade II and 55% of tumours grade III. The expression of metallothionein was negatively correlated with tumour grade and inflammation score, while the Ki67 index was positively correlated with tumour grade, inflammation score and necrosis score.

**Conclusion:**

The downregulation of MT expression in feline injection site fibrosarcomas seems to be connected with an increase in the inflammatory infiltration, hence tumour progression. This is the first study describing metallothionein expression in feline injection site fibrosarcomas.

**Supplementary Information:**

The online version contains supplementary material available at 10.1186/s12917-023-03604-5.

## Background

Feline injection site sarcoma (FISS) is a malignant tumour arising in the area of a former injection site [1, 2]. The vast majority of FISSs are fibrosarcomas, however other malignancies such as osteosarcoma, chondrosarcoma, rhabdomyosarcoma, malignant fibrous histiocytoma and myofibroblastic sarcoma have also been reported [3]. Although the first cases of FISS in cats were related to the increased use of rabies and feline leukaemia virus (FeLV) vaccinations [4–6], subsequent observations revealed that other non vaccine injectables, i.e. drugs, foreign material, microchip implantation and trauma can also induce chronic inflammation, essential in the pathogenesis of FISS [2, 7–9]. However, chronic inflammation alone probably cannot induce neoplastic transformation [8, 10]. The exact pathogenesis of FISS is not fully understood and requires further research.

Metallothioneins (MTs; with the best characterised isoforms MT I and II) are a group of small cysteine-rich proteins, playing an important role in metal homeostasis and protection against heavy metal toxicity, DNA damage, oxidative stress, and apoptosis [11]. MT I-II also shows immunomodulatory effects by reducing the synthesis of pro-inflammatory cytokines and increasing the synthesis of anti-inflammatory cytokines [12]. These properties make MT I-II an interesting object of research, particularly in tumours arising in the background of chronic inflammation, irritation or injury. Due to its antioxidant and cytoprotective role, MT I-II expression can be responsible for tumour resistance to radiotherapy or chemotherapy [12–15]. As radiotherapy and – to a lesser extent – adjuvant chemotherapy, in addition to aggressive surgery, are used in treatment of FISS [16], the expression of MT I-II could be the potential predictive factor. Furthermore, as the chronic inflammation predispose to FISS, the possible downregulation of MT I-II (due to its antiinflammatory properties) could also play a role in the pathogenesis of FISS. In feline mammary gland tumours and cutaneous melanomas, increased expression of MT I-II was related to tumour progression [17]. The expression of metallothionein has not been previously investigated in FISS.

The aim of this study was to evaluate the expression of MT I-II in feline injection site fibrosarcomas and to investigate possible relationships between the expression of metallothionein, Ki67 index, inflammation score, necrosis score and tumour grade.

## Results

Histological analysis of evaluated tumours is summarised in Table 1, and detailed characteristics of all evaluated tumours are presented in Supplementary Table 1. Five tumours were graded I (12.5%), 15 tumours—II (37.5%) and 20 tumours—III (50%). In tumours grade I, necrosis was either absent or involved less than 50% of the tumour parenchyma. Inflammatory infiltrates were absent or mild. In tumours grade II, necrosis was observed in only 40% of cases, and inflammatory infiltrates were variable. In tumours grade III, necrosis was observed in all cases, most of which involved more than 50% of the tumour parenchyma. Inflammatory infiltrates were massive in 75% of cases.

**Table 1:** Percentage of tumours grade I, II and III with particular mitotic, necrosis and inflammation scores, and IRS for MT expression (range, mean with standard deviation)

		Tumour grade		
		I	II	III
Mitotic score	1	80%	26.67%	-
	2	20%	26.67%	20%
	3	-	46.67%	80%
Necrosis score	0	60%	20%	-
	1	40%	80%	75%
	2	-	-	25%
Inflammation score	1	100%	46.67%	15%
	2	-	13.33%	10%
	3	-	40%	75%
IRS for MT expression	Range	2–9	0–6	0–6
	mean, SD	6.2 ± 3.03	1.87 ± 2.1	1.65 ± 2.18

**Fig. 1:**
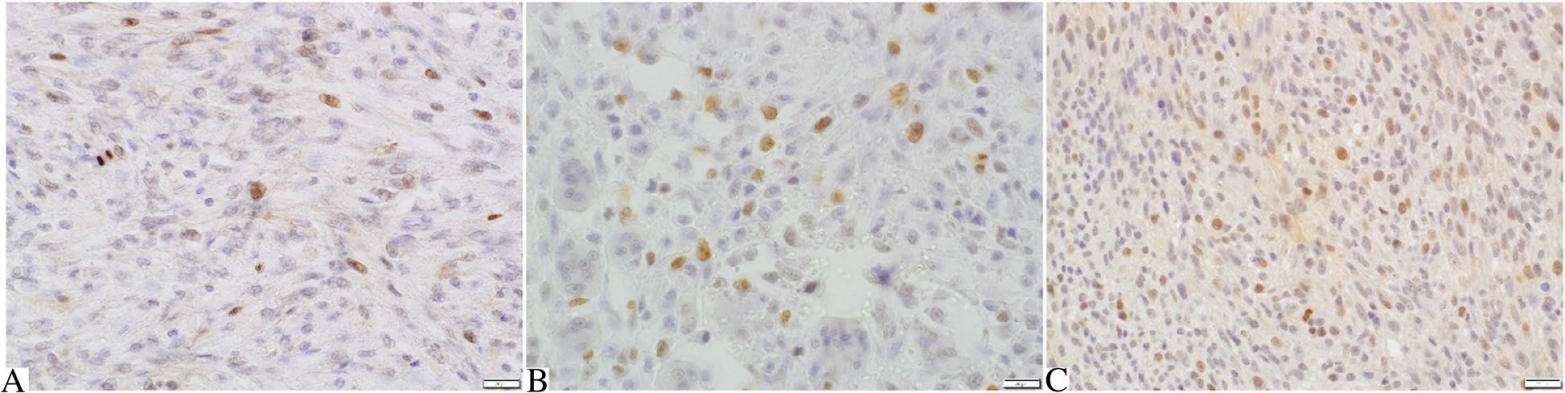
Feline injection site fibrosarcomas. **A** Tumour grade I. Small number of tumour cells show nuclear expression of Ki67. **B** Tumour grade II. Moderately numerous tumour cells show nuclear expression of Ki67. **C** Tumour grade III. Numerous tumour cells show nuclear expression of Ki67. Ki67 immunostaining with DAB, counterstaining with Mayer’s haematoxylin, 400x

**Fig. 2:**
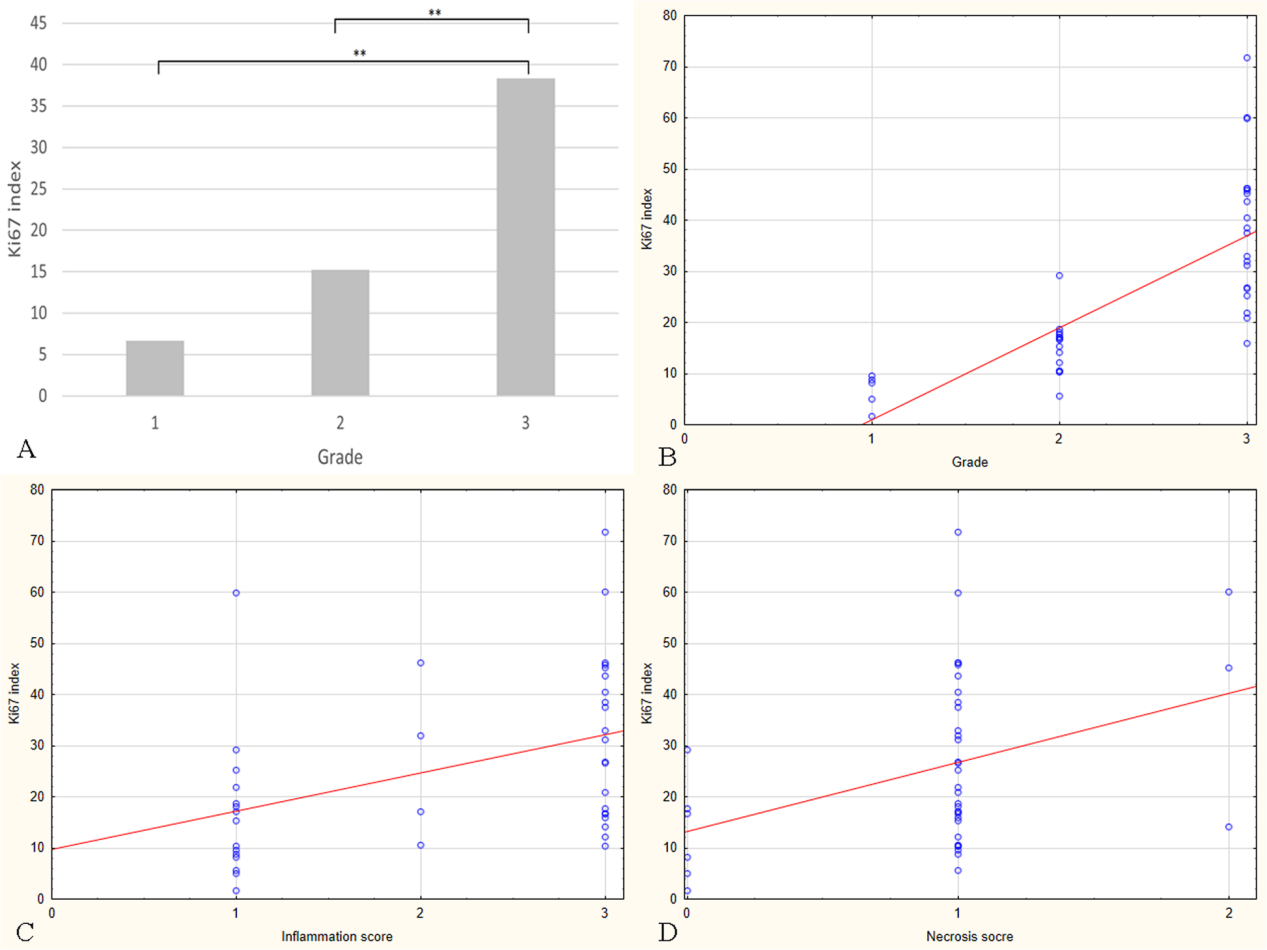
Feline injection site fibrosarcomas. **A** Mean Ki67 index values in tumours grade I, II, and III. The Ki67 index was significantly higher in tumours grade III compared to grade II (*p* = 0.000) and I (*p* = 0.000). **B** Positive correlation between the Ki67 index and the tumour grade. The Ki67 index increased with the tumour grade (*r* = 0.853; *p* = 0.000). **C** Positive correlation between the Ki67 index and the inflammation score. The Ki67 index increased with the inflammation score (*r* = 0.452; *p* = 0.003). **D** Positive correlation between the Ki67 index and the necrosis score. The Ki67 index increases with the necrosis score (*r* = 0.37; *p* = 0.018)

The mean Ki67 (Fig. 1A-C) index was 6.64 ± 3.31 in tumours grade I, 15.25 ± 5.36 in tumours grade II and 38.4 ± 14.4 in tumours grade III. Statistical analysis revealed that the Ki67 index was significantly higher in tumours grade III compared to grade II (*p* = 0.000) and to grade I (*p* = 0.000; Fig. 2A). Additionally, the Ki67 index was positively correlated with tumour grade (*r* = 0.853; *p* = 0.000; Fig. 2B), inflammation score (*r* = 0.452; *p* = 0.003; Fig. 2C), and necrosis score (*r* = 0.37; *p* = 0.018; Fig. 2D).

**Fig. 3:**
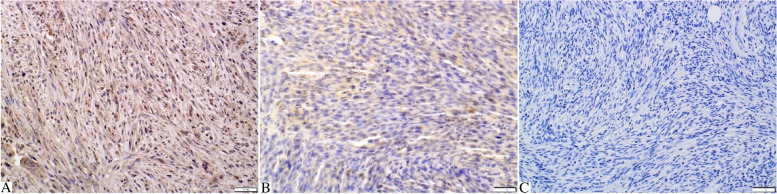
Feline injection site fibrosarcomas. **A** Tumour grade I. Numerous tumour cells (quantitative score: 3) show moderate (qualitative score: 2) expression of metallothionein (IRS: 6). The expression is mostly cytoplasmic, but the nuclear expression is also present in some tumour cells. **B** Tumour grade II. Moderately numerous tumour cells (quantitative score: 2) show weak (qualitative score: 1) cytoplasmic expression of metallothionein (IRS: 2). **C** Tumour grade III. Absence of metallothionein expression in tumour cells. Metallothionein immunostaining with DAB, counterstaining with Mayer’s haematoxylin, 200x

**Fig. 4:**
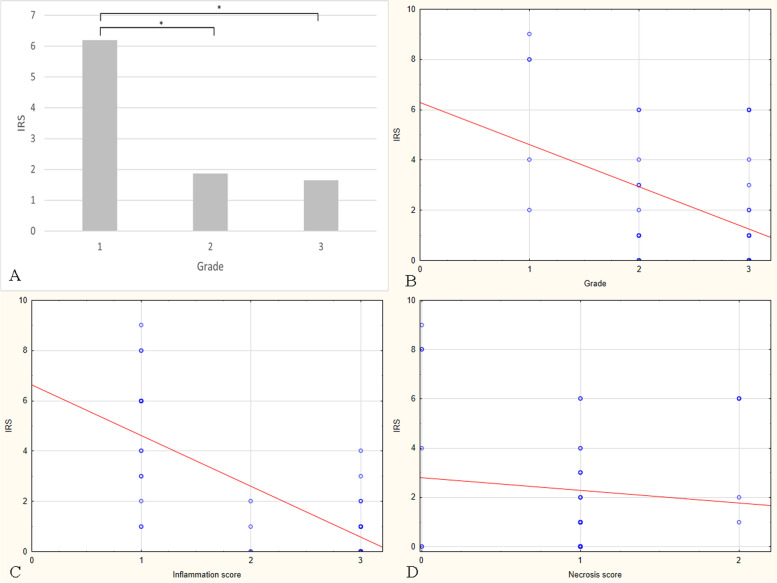
Feline injection site fibrosarcomas. **A** Mean IRS for MT expression values in tumours grade I, II, and III. The IRS for MT expression was significantly higher in tumours grade I compared to grade II (*p* = 0.04) and III (*p* = 0.012). **B** Negative correlation between the IRS for MT expression and the tumour grade. The metallothionein expression decreased as the tumour grade was higher (*r* = -0.356; *p* = 0.024). **C** Negative correlation between the IRS for MT expression and the inflammation score. The metallothionein expression decreased as the inflammation score was higher (*r* = -0.713; *p* = 0.000). **D** No correlation was found between IRS for MT expression and necrosis score (*r* = 0.014; *p* = 0.9)

The cytoplasmic and sometimes nuclear MT expression was observed in all tumours grade I (100%), 10 tumours grade II (66.67%), and 11 tumours grade III (55%) (Fig. 3A-C). The mean immunoreactive score (IRS) for MT expression was 6.2 ± 3.03, 1.87 ± 2.1, and 1.65 ± 2.18 for tumours grade I, II, and III, respectively. Statistical analysis showed that the IRS for MT expression was significantly higher in grade I compared to grade II (*p* = 0.04) and to grade III (*p* = 0.012; Fig. 4A). Moreover, IRS for MT expression was negatively correlated with tumour grade (*r* = -0.356; *p* = 0.024; Fig. 4B) and inflammation score (*r *= -0.713; *p* = 0.000; Fig. 4C). No correlation was found between IRS for MT expression and necrosis score (*r* = 0.014; *p* = 0.9; Fig. 4D).

## Discussion

We observed the expression of MT in 26 cases (out of 40) of feline injection site fibrosarcomas, and this expression was negatively correlated with the degree of inflammation and tumour grade. It was previously shown that MT has immunomodulatory properties, suppressing T-dependent humoral immune response [18] and function of cytotoxic T-cells [19]. The chronic inflammation is a key point in the pathogenesis of FISS. Free radicals generated during persistent inflammation cause DNA damage [20]. Furthermore, the degree of inflammatory infiltration is one of three parameters of a recently proposed grading system for feline soft tissue sarcomas [21]. Therefore, the negative correlation between MT expression and the degree of inflammatory infiltration (and tumour grade) in feline injection site fibrosarcomas indicates that the downregulation of MT expression may be involved in tumour progression. The downregulation of MT expression was observed in several human cancers, i.e. liver, colon and prostate [22–24]. In human colon carcinoma downregulation of a specific isoform of MT (MT1F) resulted from loss of heterozygosity [24]. As MT protects cells from oxidative stress, downregulation of MT expression might be linked to increased risk of DNA damage and mutagenic changes, responsible for carcinogenesis [17, 25] which is also very likely in feline injection site fibrosarcoma. On the contrary, a positive correlation between MT expression and tumour grade was found in selected human soft tissue sarcomas [26]. The elevated expression of MT allows tumour cells to evade the host immune response [19]. Furthermore, as the MT is responsible for resistance to radiotherapy [25], it can be assumed that feline injection site fibrosarcomas grade I could be possibly more resistant to radiotherapy (compared to grade II and III), but this relationship must be further evaluated in prospective clinical studies. Although MT expression was mainly cytoplasmic, we also observed nuclear expression in some tumour cells. The nuclear localization of MT is transiently observed during cell proliferation and early differentiation; this protein protects DNA from oxidative damage and apoptosis [27]. It was previously shown that nuclear expression of MT was responsible for cisplatin resistance in ovarian cancer [28].

The Ki67 index is associated with tumour proliferation, growth, and progression, and it is commonly used in human and veterinary medicine as a prognostic marker [29–33]. A high Ki67 index is associated with poor prognosis in several human and animal tumours, e.g., human and feline mammary gland tumours and human soft-tissue sarcomas [29, 34, 35]. In our study, the Ki67 index was positively correlated with tumour grade, but its prognostic significance must be elucidated in the further studies. Furthermore, we found a positive correlation between Ki67 index and inflammatory score. In a study on canine colorectal carcinoma, Ki67 index was positively correlated with the number of tumour associated macrophages and mast cells [36]. It is suggested that inflammatory cells have an impact on cancer development by increasing the invasive capacity, angiogenesis and motility of tumour cells [37]. In human breast cancer, the inflammation is closely associated with tumour proliferation and progression. This correlation may reflect an active immune response to poor tumour cell differentiation or may be a consequence of increased cytokine secretion by high-grade tumours [38]. The association between inflammatory infiltration and Ki67 index (as well as tumour grade) in feline injection site fibrosarcomas suggests that the inflammation plays an important role not only in pathogenesis, but also in tumour progression. Additionally, we found a positive correlation between Ki67 index and necrosis score. This is not surprising since tumour necrosis is a consequence of ischemic injury caused by rapid tumour growth [39]. Furthermore, necrotic cells release factors enhancing tumour cell proliferation [40]. In human breast cancer, the degree of the tumour necrosis reflects higher metabolic activity and more rapid growth, which results in higher Ki67 index in tumours with extent areas of necrosis [41].

## Conclusion

The downregulation of MT expression in feline injection site fibrosarcomas is connected with an increase in the inflammatory infiltration. Both the reduction of MT expression and the increased degree of inflammatory infiltration seem to be associated with tumour progression.

## Methods

### Study design

The study included surgically excised cutaneous tumours collected from 40 cats between 2019–2020, 28 (70%) males and 12 (30%) females, aged 4–17 (mean: 11 ± 3 years). The samples were fixed in 10% neutral buffered formalin, processed routinely, embedded in paraffin wax, cut and stained with Mayer’s haematoxylin and eosin (HE). Inclusion criteria included morphological features [42, 43] and localisation in the injection site: interscapular region (50%; 20/40 cases), thigh (12.5%; 5/40 cases), flank (12.5%; 5/40 cases), lumbar area (10%; 4/40 cases), shoulder (10%; 4/40 cases), and dorsal area of the neck (5%; 2/40 cases). Histologically, the tumours were graded into grade I, II and III, according to the grading system proposed by Dobromylskyj et al. [21] for feline soft tissue sarcomas (Table 2). The number of inflammatory cells was categorised as none or mild (1), when inflammatory cells were absent or only few, scattered throughout the tumour parenchyma; moderate (2), when inflammatory cells were more numerous, forming distinct foci within the tumour parenchyma; and severe (3), when the inflammatory cells were numerous, and formed large foci. The necrosis extent was classified as 0 (no necrosis within the tumour), 1 (necrotic area up to 50% of tumour parenchyma) and 2 (necrotic area covers more than 50% of tumour parenchyma).

**Table 2:** Grading system for cutaneous soft tissue sarcomas [21]

**Mitotic score**	**Number of mitoses in 2.37mm**^**2**^**; 400x**
1	0–9
2	10–19
3	> 19
**Tumour necrosis score**	**Necrosis as % of tumour area**
0	Absent
1	≤ 50%
2	> 50%
**Inflammation score**	
1	None or mild
2	Mild to moderate
3	Severe

Histological grade (Sum of score)

≤ 3 –I (low grade)

4 or 5 – II (intermediate grade)

6 or more – III (high grade)

### Immunohistochemical evaluation

The sections for immunohistochemistry were mounted on silanized glass slides. Heat-induced antigen retrieval was performed in Tris–EDTA buffer pH 9.0 (EnVision™ Flex Target Retrieval Solution High pH, DAKO, Glostrup, Denmark) for 20 min at 96 °C using a PT-Link module (Dako, Glostrup, Denmark). Immunohistochemical examination of each tumour was performed using primary antibodies: Ki67 (monoclonal mouse anti-human, clone MIB-1, dilution 1:75, incubation time: 30 min in a humid chamber at room temperature; Dako, Glostrup, Denmark) and metallothionein (monoclonal mouse anti-metallothionein I-II, clone UC1MT, dilution 1:1000, incubation time: 60 min in a humid chamber at 60℃; Abcam, Cambridge, Great Britain) and a visualisation system based on the immunoperoxidase method, with 3,3-diaminobenzidine (DAB) as a substrate (EnVision + System-HRP, Mouse, Dako, Glostrup, Denmark). The slides were counterstained with Mayer’s haematoxylin. Positive and negative control slides were processed together with the evaluated slides (metallothionein – feline mammary gland, Ki67 – feline skin). Brown precipitate at the antigen site was regarded as a positive reaction (Ki67 – nuclear; MT I-II – nuclear and cytoplasmic). The slides were evaluated under a light microscope (BX63, Olympus, Tokyo, Japan) using CellSense (Olympus, Tokyo, Japan) software.

### Ki67 evaluation

The expression of Ki67 was calculated in 10 HPFs (high power fields, 400x), in areas with the most numerous positive cells (“hot-spots”), avoiding areas of inflammation and necrosis The Ki67 index was expressed as a percentage of positive cells and was obtained by calculating the ratio of positively stained tumour cells and all tumour cells observed in the analysed microscopic fields, as was previously described by Patruno et al. [32]. Mean values (with standard deviation) were calculated for groups with grades I, II and III.

### MT evaluation

The expression of MT was assessed in 10 randomly selected HPFs (400x), using a semiquantitative scale that included the intensity of the immunostaining (0: none; 1: weak; 2: moderate; 3: intense) and the percentage of immunoreactive tumour cells (1: < 10%; 2: 10–50%; 3: 51–80%; 4: > 80%). IRS for MT expression was a product of multiplication of points given for individual traits and ranged between 0 and 12 [44–47]. Mean values (with standard deviation) were calculated for groups with grades I, II and III.

### Statistical analysis

The variables were checked for normality using the Shapiro–Wilk test. Differences in Ki67 index and IRS for MT expression between groups with grade I, II and III were analysed using Kruskal‒Wallis H test (one-way ANOVA on ranks) followed by Dunn’s post-hoc test. Correlations between tumour grade, inflammation score, necrosis score, Ki67 index and IRS for MT expression were examined using Spearman’s rank correlation (r: Spearman’s rank correlation coefficient); *p* < 0.05 was considered statistically significant, and *p* < 0.001 as highly significant. The statistical analysis was performed using Statistica 14 software (StatSoft Inc., Tulsa, OK, USA).

## Supplementary Information

**Additional file 1:** **Supplementary Table 1.** Detailed characteristics of evaluated feline injection site fibrosarcomas.

## Data Availability

Data used in this study are available from the corresponding author on reasonable request.
