# Supplementary material for: Metallothionein expression in feline injection site fibrosarcomas
Source: BMC Vet Res. 2023 Feb 10;19:42. doi: 10.1186/s12917-023-03604-5 (PMC9912506; doi:10.1186/s12917-023-03604-5)
Supplement: Supplementary file 1 — Additional file 1: Supplementary Table 1. Detailed characteristics of evaluated feline injection site fibrosarcomas. [file 12917_2023_3604_MOESM1_ESM.docx]

| Case no. | Mitotic score | Necrosis score | Inflammation score | IRS for MT expression | Histological grade |
| --- | --- | --- | --- | --- | --- |
| 1 | 1 | 0 | 1 | 4 | I |
| 2 | 2 | 0 | 1 | 9 | I |
| 3 | 1 | 1 | 1 | 8 | I |
| 4 | 1 | 1 | 1 | 8 | I |
| 5 | 1 | 0 | 1 | 2 | I |
| 6 | 3 | 1 | 1 | 6 | II |
| 7 | 3 | 1 | 1 | 1 | II |
| 8 | 1 | 1 | 3 | 0 | II |
| 9 | 3 | 1 | 1 | 3 | II |
| 10 | 2 | 1 | 2 | 2 | II |
| 11 | 1 | 1 | 3 | 0 | II |
| 12 | 2 | 1 | 2 | 0 | II |
| 13 | 2 | 0 | 3 | 1 | II |
| 14 | 1 | 1 | 3 | 1 | II |
| 15 | 3 | 1 | 1 | 1 | II |
| 16 | 3 | 1 | 1 | 3 | II |
| 17 | 1 | 1 | 3 | 0 | II |
| 18 | 3 | 0 | 1 | 6 | II |
| 19 | 2 | 0 | 3 | 0 | II |
| 20 | 3 | 1 | 1 | 4 | II |
| 21 | 3 | 1 | 3 | 0 | III |
| 22 | 3 | 1 | 3 | 4 | III |
| 23 | 3 | 1 | 3 | 1 | III |
| 24 | 3 | 2 | 1 | 6 | III |
| 25 | 3 | 1 | 3 | 0 | III |
| 26 | 3 | 1 | 3 | 0 | III |
| 27 | 3 | 1 | 3 | 0 | III |
| 28 | 3 | 1 | 3 | 0 | III |
| 29 | 3 | 2 | 1 | 6 | III |
| 30 | 3 | 1 | 3 | 2 | III |
| 31 | 3 | 2 | 1 | 6 | III |
| 32 | 2 | 2 | 3 | 3 | III |
| 33 | 3 | 1 | 2 | 0 | III |
| 34 | 3 | 1 | 3 | 2 | III |
| 35 | 3 | 1 | 3 | 1 | III |
| 36 | 3 | 1 | 2 | 1 | III |
| 37 | 2 | 1 | 3 | 1 | III |
| 38 | 3 | 1 | 3 | 0 | III |
| 39 | 2 | 2 | 3 | 0 | III |
| 40 | 2 | 1 | 3 | 0 | III |

Supplementary Table 1. Detailed characteristics of evaluated feline injection site fibrosarcomas.
